# Supplementary material for: A vegetable fat-based diet delays psychomotor and cognitive development compared with maternal dairy fat intake in infant gray mouse lemurs
Source: Commun Biol. 2024 May 20;7:609. doi: 10.1038/s42003-024-06255-w (PMC11106064; doi:10.1038/s42003-024-06255-w)

Supplementary Table 1: Offspring body mass across the first 5 days of life, expressed as median ± IQR (sample size). Statistical tests rely on mixed linear models. Not all animals were weighted at each date, since sometimes the weighting date fell on the week-end with no staff on the site, hence contributing to variation in the sample size.

		D+1	D+3	D+5	Effect of diet
Diet	DF	6.70 ± 1.15 (23)	7.90 ± 2.80 (23)	9.15 ± 4.00 (16)	*p=0.95*
	VF	6.75 ± 2.25 (22)	9.60 ± 1.75 (12)	10.75 ± 1.33 (12)	
Effect of Date			*p<0.001*	*p<0.001*	
Effect of Diet:Date			*p=0.37*	*p=0.28*	

Supplementary Table 2: Offspring's characteristics. Gestation length and sex ratio have been tested using a mixed linear model and Fisher's exact test for count data respectively. Gestation length has been estimated by the duration between the first oestrus appearance and the date of birth. It must be brought to attention that the oestrus can last 3 days, hence bringing some uncertainties about the gestation length estimation. [1]: One DF female had initially four pups but ended up with only three since the fourth one died at birth; [2]: Two VF females had initially two pups (litter size = 2) but ended up with only one pup since the other one died at birth.

		Litter size			Total number of litters	Gestation length in days (median ± IQR)		Sex ratio	
		1 infant	2 infants	3 infants					
Diet	DF	2	4	5 (4)[1]	11	61.00 ± 1.50	*N.S. p=0.23*	1.27	*N.S. p=0.15*
	VF	5 (3)[2]	1 (3)[2]	6	12	62.50 ± 4.25		0.47	
Total		7 (5)[2]	5 (8)[2]	11 (10)[1]	23	61.00		0.79	

Supplementary Table 3: Number (and proportion) of animals that failed at the negative geotaxis task at D+8, *i.e.*, that fell or did not reach the marks within the three trials and were therefore excluded from further analysis among successful animals.

	Horizontal mark	Vertical mark
DF	4/25<br>(16%)	8/25<br>(32%)
VF	12/24<br>(50%)	15/24<br>(62.5%)

Supplementary Table 4: Number (and proportion) of animals that failed at the horizontal rod task at D+8, *i.e.*, that fell or did not reach the nest box, at each level of difficulty and were therefore excluded from further analysis among successful animals.

	Distance from the nest-box		
	11 cm	22 cm	33 cm
DF	2/25<br>(8%)	4/24<br>(16.7%)	5/24<br>(20.8%)
VF	9/23<br>(39.1%)	11/24<br>(45.8%)	13/22<br>(59.1%)

Supplementary Table 5: Requirements of total diet and composition in macronutrients and energy

		DF (g)	VF (g)	Lipids (g)	Carbohydrates (g)	Proteins (g)
Mixture	*Experimental lipidic input*	60.00	50.00	50.00	0.00	0.00
	Including water	10.00	0.00	0.00	0.00	0.00
	Blédine©	46.75		0.65	39.73	4.96
	Gingerbread	27.27		0.33	19.63	0.95
	Lipid-free dairy proteins	100.00		0.00	52.00	35.00
	Lipid-free white cheese	142.85		<0.71	6.71	10.71
	Egg yolk	8.00		1.89	0.02	1.10
	Water	320.00	330.00	0.00	0.00	0.00
Fruits	*Royal Gala Apple slice*	330.20	330.20	0.83	39.29	0.83
	Banana slice	165.10	165.10	0.83	32.52	1.75
	Cucumber slice	165.10	165.10	0.18	4.19	1.06
Total mass diet (g)		1375.27		55.41	194.10	56.35
Total mass diet (%)		305.87		18.12	63.46	18.42
Energy content (%Kcal)		100.00		33.24	51.74	15.02

Supplementary Table 6: Origin of the experimental lipidic input

	DF (g)	VF (g)
Butter	45 (including 10 of water)	0
Palm oil	0	32.5
Oleic acid-rich sunflower oil	10	12.5
Rapeseed oil	5	5
Total mass input	60 (including 10 of water)	50

Supplementary Table 7: Fatty acid composition of the total diet (fruits and vegetables excluded), expressed as the mean ± standard error.

Common name	Nomenclature	DF (%) (n=6)	VF (%) (n=6)
Saturated fatty acids		49.06 ± 0.23	34.68 ± 0.17
Caproic acid	*C6:0*	1.18 ± 0.04	0.00 ± 0.00
Caprylic acid	*C8:0*	1.02 ± 0.02	0.00 ± 0.00
Capric acid	*C10:0*	2.59 ± 0.03	0.00 ± 0.00
Lauric acid	*C12:0*	3.10 ± 0.02	0.08 ± 0.03
Myristic acid	*C14:0*	8.89 ± 0.03	0.84 ± 0.01
Short- and medium chain- fatty acids	*C4:0 – C14:0*	16.79 ± 0.14	0.92 ± 0.03
Pentadecanoic acid	*C15:0*	0.97 ± 0.00	0.00 ± 0.00
Palmitic acid	*C16:0*	22.55 ± 0.09	29.70 ± 0.17
Stearic acid	*C18:0*	8.75 ± 0.04	4.06 ± 0.01
Arachidic acid	*C20:0*	0.00 ± 0.00	0.35 ± 0.00
Monounsaturated fatty acids		42.53 ± 0.16	52.18 ± 0.12
Myristoleic acid	C14:1	0.77 ± 0.01	0.00 ± 0.00
Palmitoleic acid	C16:1	0.29 ± 0.00	0.26 ± 0.00

Margaroleic acid	C17:1	0.12 ± 0.04	0.00 ± 0.00
Trans-vaccenic acid	C18:1 t n-7	2.40 ± 0.05	0.00 ± 0.00
Vaccenic acid	C18:1 n-7	0.88 ± 0.01	1.03 ± 0.01
Elaidic acid	C18:1 t n-9	0.21 ± 0.07	0.00 ± 0.00
Oleic acid	C18:1 n-9	37.51 ± 0.14	50.88 ± 0.12
Polyunsaturated fatty acids		8.41 ± 0.09	12.56 ± 0.03
Linoleic acid (LA)	*C18:2 n-6*	6.58 ± 0.05	11.53 ± 0.03
α-linolenic acid (ALA)	*C18:3 n-3*	1.53 ± 0.01	1.03 ± 0.00
LA:ALA ratio		4.29 ± 0.01	11.25 ± 0.02

Supplementary Figure 1: Nutritional follow-up and psychomotor tasks in relation to the timeline (inserts A to D)

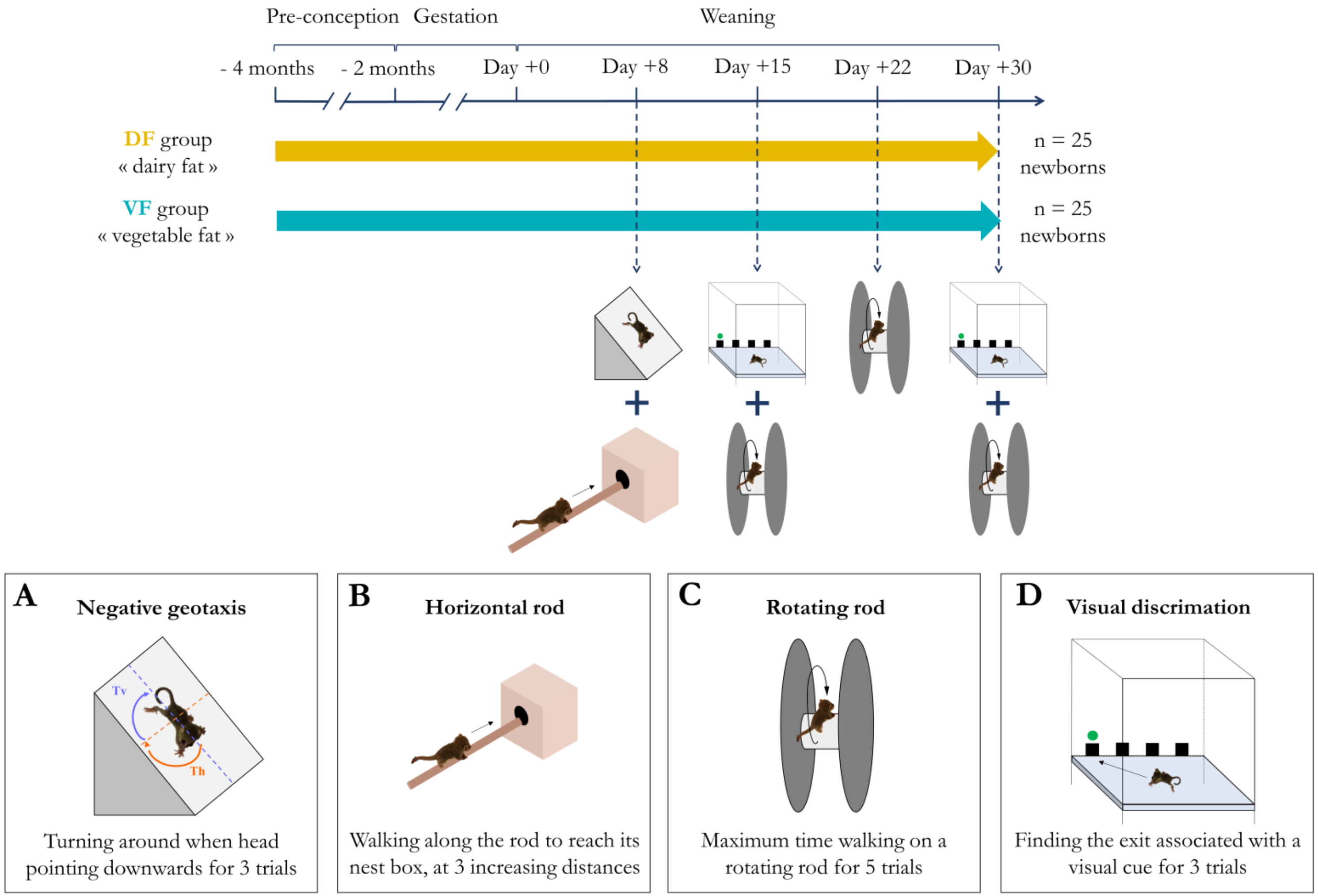

Supplement: Supplementary file 2 — Supplementary Information [file 42003_2024_6255_MOESM2_ESM.pdf]
