# Supplementary material for: A vegetable fat-based diet delays psychomotor and cognitive development compared with maternal dairy fat intake in infant gray mouse lemurs
Source: Commun Biol. 2024 May 20;7:609. doi: 10.1038/s42003-024-06255-w (PMC11106064; doi:10.1038/s42003-024-06255-w)
Supplement: Supplementary file 3 — Description of Supplementary Materials [file 42003_2024_6255_MOESM3_ESM.docx]

**Description of Additional Supplementary Files**

**File name:** Supplementary Data
**Description:** The source data behind the graphs in the paper.
