# Supplementary material for: A vegetable fat-based diet delays psychomotor and cognitive development compared with maternal dairy fat intake in infant gray mouse lemurs
Source: Commun Biol. 2024 May 20;7:609. doi: 10.1038/s42003-024-06255-w (PMC11106064; doi:10.1038/s42003-024-06255-w)
Supplement: Supplementary file 5 — Reporting Summary [file 42003_2024_6255_MOESM5_ESM.pdf]

nature portfolio

Corresponding author(s): YOHANN CHAUDRON, FABIEN PIFFERI

Last updated by author(s): Apr 13, 2024

Reporting Summary

Nature Portfolio wishes to improve the reproducibility of the work that we publish. This form provides structure for consistency and transparency in reporting. For further information on Nature Portfolio policies, see our Editorial Policies and the Editorial Policy Checklist.

Statistics

For all statistical analyses, confirm that the following items are present in the figure legend, table legend, main text, or Methods section.

n/a	Confirmed	
☐	☒	The exact sample size (*n*) for each experimental group/condition, given as a discrete number and unit of measurement
☐	☒	A statement on whether measurements were taken from distinct samples or whether the same sample was measured repeatedly
☐	☒	The statistical test(s) used AND whether they are one- or two-sided<br>*Only common tests should be described solely by name; describe more complex techniques in the Methods section.*
☐	☒	A description of all covariates tested
☐	☒	A description of any assumptions or corrections, such as tests of normality and adjustment for multiple comparisons
☐	☒	A full description of the statistical parameters including central tendency (e.g. means) or other basic estimates (e.g. regression coefficient) AND variation (e.g. standard deviation) or associated estimates of uncertainty (e.g. confidence intervals)
☐	☒	For null hypothesis testing, the test statistic (e.g. *F*, *t*, *r*) with confidence intervals, effect sizes, degrees of freedom and *P* value noted<br>*Give P values as exact values whenever suitable.*
☒	☐	For Bayesian analysis, information on the choice of priors and Markov chain Monte Carlo settings
☐	☒	For hierarchical and complex designs, identification of the appropriate level for tests and full reporting of outcomes
☐	☒	Estimates of effect sizes (e.g. Cohen's *d*, Pearson's *r*), indicating how they were calculated

Our web collection on statistics for biologists contains articles on many of the points above.

Software and code

Policy information about availability of computer code

Data collection	All trials were videotaped with a GoPro HERO4 (GoPro, Inc. CA, USA). Videos were analysed with Noldus EthoVision XT software.
Data analysis	All statistical analyses were conducted using R (4.2.2 version) software.

For manuscripts utilizing custom algorithms or software that are central to the research but not yet described in published literature, software must be made available to editors and reviewers. We strongly encourage code deposition in a community repository (e.g. GitHub). See the Nature Portfolio guidelines for submitting code & software for further information.

Data

Policy information about availability of data

All manuscripts must include a data availability statement. This statement should provide the following information, where applicable:

- Accession codes, unique identifiers, or web links for publicly available datasets
- A description of any restrictions on data availability
- For clinical datasets or third party data, please ensure that the statement adheres to our policy

The data that support the findings of this study are available in the Supplementary Data file.

Human research participants

Policy information about studies involving human research participants and Sex and Gender in Research.

Reporting on sex and gender	*Use the terms sex (biological attribute) and gender (shaped by social and cultural circumstances) carefully in order to avoid confusing both terms. Indicate if findings apply to only one sex or gender; describe whether sex and gender were considered in study design whether sex and/or gender was determined based on self-reporting or assigned and methods used. Provide in the source data disaggregated sex and gender data where this information has been collected, and consent has been obtained for sharing of individual-level data; provide overall numbers in this Reporting Summary. Please state if this information has not been collected. Report sex- and gender-based analyses where performed, justify reasons for lack of sex- and gender-based analysis.*
Population characteristics	*Describe the covariate-relevant population characteristics of the human research participants (e.g. age, genotypic information, past and current diagnosis and treatment categories). If you filled out the behavioural & social sciences study design questions and have nothing to add here, write "See above."*
Recruitment	*Describe how participants were recruited. Outline any potential self-selection bias or other biases that may be present and how these are likely to impact results.*
Ethics oversight	*Identify the organization(s) that approved the study protocol.*

Note that full information on the approval of the study protocol must also be provided in the manuscript.

Field-specific reporting

Please select the one below that is the best fit for your research. If you are not sure, read the appropriate sections before making your selection.

☒ Life sciences ☐ Behavioural & social sciences ☐ Ecological, evolutionary & environmental sciences

For a reference copy of the document with all sections, see nature.com/documents/nr-reporting-summary-flat.pdf

Life sciences study design

All studies must disclose on these points even when the disclosure is negative.

Sample size	Since no previous study has been led on the impact of nutrition on the psychomotor development of mouse lemurs, sample size could not be calculated a priori without leading to unreliable results. However, previously dietary interventions in adult mouse lemurs with cognitive measures enrolled between 6 and 12 animals per experiment group. Thus, since the litter is the smallest experimental unit of the present study, a sample size of 11-12 litters was determined.
Data exclusions	One video from the negative geotaxis task and eight videos from the horizontal rod task were inoperable because of investigator's misconduct regarding the protocol and were therefore excluded from the analysis.
Replication	Findings were not replicated since we used a primate model for which it is difficult ethically and time-consuming to attain a sufficient sample size (it took 2 years to attain n=50 pups).
Randomization	Multiparous female mouse lemurs (2 to 4 years old) were randomly assigned to one of both experimental groups using a computer based random function. On the same day, testing order of the animals within a litter (in the case there were twins or triplets) and between litters were randomized.
Blinding	The investigator was not blinded to the diet of the animals, since it was the same person that fed the animals and take care of them for at least one year of this 2 years-study until additional staff was recruited.

Reporting for specific materials, systems and methods

We require information from authors about some types of materials, experimental systems and methods used in many studies. Here, indicate whether each material, system or method listed is relevant to your study. If you are not sure if a list item applies to your research, read the appropriate section before selecting a response.

Materials & experimental systems

n/a	Involved in the study
☒	☐ Antibodies
☒	☐ Eukaryotic cell lines
☒	☐ Palaeontology and archaeology
☐	☒ Animals and other organisms
☒	☐ Clinical data
☒	☐ Dual use research of concern

Methods

n/a	Involved in the study
☒	☐ ChIP-seq
☒	☐ Flow cytometry
☒	☐ MRI-based neuroimaging

Animals and other research organisms

Policy information about studies involving animals; ARRIVE guidelines recommended for reporting animal research, and Sex and Gender in Research

Laboratory animals	Grey mouse lemurs (Microcebus murinus): reproductive females (2 to 4 years old) and their pups (until 30 days old) of both sex
Wild animals	The study did not involve wild animals.
Reporting on sex	Sex has been used as an explanatory variable in our models. Since no effect of sex was reported, and this variable increased the AICc, it was removed from the selected models. We did not find a bias in the sex ratio according to diet groups (see Supplementary Table B).
Field-collected samples	The study did not involve samples collected from the field.
Ethics oversight	All experiments were performed in accordance with the Principles of Laboratory Animal Care (National Institutes of Health publication 86–23, revised 1985) and the European Communities Council Directive (86/609/EEC). The research was conducted under the approval of the Cuvier Ethical Committee (Committee number 68 of the ‘Comité National de Réflexion Ethique sur l’Expérimentation Animale’) under authorization number # 29784-2021021215198648 v3.

Note that full information on the approval of the study protocol must also be provided in the manuscript.
